# Perioperative Pain Management Strategies and Postoperative Outcomes in Tonsillectomy Patients Aged Seven to 14 Years: A Single-Center Study

**DOI:** 10.7759/cureus.108504

**Published:** 2026-05-08

**Authors:** Afnan Amjad

**Affiliations:** 1 Anaesthesia and Critical Care, Naas General Hospital, Kildare, IRL; 2 Anaesthesia and Critical Care, Lady Reading Hospital, Peshawar, PAK

**Keywords:** acetaminophen, analgesia, pediatric pain management, postoperative outcomes, tonsillectomy

## Abstract

Background

Tonsillectomy is a common pediatric procedure requiring effective perioperative pain management to minimize complications and promote recovery. In resource-limited settings, standardized protocols are often challenged by infrastructural constraints and variable adherence to multimodal analgesia guidelines, risking suboptimal pain control and delayed discharge.

Objective

To evaluate a standardized acetaminophen-based pain management protocol and its impact on postoperative outcomes in children aged seven to 14 years undergoing tonsillectomy at the Lady Reading Hospital, a tertiary care hospital in Peshawar, Khyber Pakhtunkhwa, Pakistan.

Methods

A prospective observational study was conducted at Lady Reading Hospital, Peshawar, between October 2024 and July 2025. Data from 99 consecutively enrolled patients were collected using a structured proforma, capturing demographics, perioperative medications, pain scores (Numeric Rating Scale 0-10 or Wong-Baker Faces scale), discharge status, oral intake tolerance, and pain at discharge. The analgesic protocol was primarily acetaminophen-based. Patients received intraoperative acetaminophen, with ibuprofen administered postoperatively as needed for breakthrough pain. A single dose of tramadol was given at induction as part of the standard anesthesia protocol. Descriptive statistics were analyzed using SPSS version 25. Ethical approval was obtained from the hospital research committee.

Results

All 99 patients (100%) received uniform intra-/post-operative acetaminophen (15 mg/kg). Adjunct ibuprofen, as rescue analgesia (PRN), was administered to only six out 99 (6.1%) of patients, whose pain did not subside. Postoperative pain scores revealed that 73.7% (73/99) of the patients reported mild pain (score=3 on the 0-10 pain scale), while 20.2% (20/99) of the patients reported moderate pain (score=5 on the 0-10 pain scale), with the latter predominantly observed in younger children (seven to nine years). All patients 99/99 (100%) tolerated cold liquids orally. Although no patient (0/99) reported pain at discharge (assessed as a binary Yes/No field by nursing staff at time of discharge; not a formal repeated pain score), while 73.7% (73/99) were discharged home after 24 hours, with retention rates peaking among 26/99 (26.3%) patients aged 11-year-olds.

Conclusion

A standardized perioperative acetaminophen-based monotherapy was associated with generally low pain scores and favorable recovery outcomes, including high rates of oral intake tolerance and minimal pain at discharge in this cohort. However, a proportion of patients still experienced moderate pain, which may be related to the limited use of ibuprofen. Given the observational design and absence of a control group, these findings should be interpreted with caution. Further controlled studies are needed to compare analgesic strategies and to determine the optimal role of adjuncts such as ibuprofen and age-specific dosing. Additionally, system-level factors influencing timely discharge warrant further investigation.

## Introduction

Worldwide, tonsillectomy is regarded as one of the most frequent surgeries performed in pediatric patients under the age of 15 years [1]. Though effective for treating recurrent infections and obstructive sleep apnea, tonsillectomy invariably results in significant postoperative pain that affects recovery, prolongs hospital stays, and reduces quality of life during healing [2]. Moreover, in pediatric patients under the age of 15 years, this pain is experienced severely because of developmental differences in pain perception, metabolism of analgesics, and psychological coping mechanisms, giving rise to complex challenges for clinicians [3].

In resource-constrained settings like Pakistan, recommended protocols for perioperative analgesia are often ignored or poorly implemented. This gap results in heterogeneous practices in which despite the risks of respiratory distress, opioids overdose is relied upon as a rescue therapy. Not only that but cultural barriers such as parental concerns of medication side effects and limited health literacy often result in underdosing of first-line analgesics [4]. Existing international guidelines such as American Pain Society (APS) and American Society of Anesthesiologists (ASA) strongly recommend the practice of multimodal analgesia, combining acetaminophen with non-steroidal anti-inflammatory drugs (NSAIDs) like ibuprofen, to produce synergistic effects, reduce over-reliance on opioids, and expedite recovery. This is further supported by evidence, which confirms the efficacy of this approach in the decrease of pain scores by 30%-40% and shortening of hospital stays by 1.2 days on average compared to monotherapy [5].

However, based on the 2024 study conducted at Hayatabad Medical Complex, Peshawar, ibuprofen alone failed to adequately manage post-tonsillectomy pain, as none of the children (0/136) achieved a pain-free state (VAS 1-3) with ibuprofen alone, whereas 23.4% of children receiving combination therapy reported no pain [6]. This is concerning given that ibuprofen’s anti-inflammatory properties specifically target surgical tissue trauma, while acetaminophen acts centrally on pain pathways. This study bridges critical evidence gaps by evaluating a standardized acetaminophen-based protocol at Lady Reading Hospital, a high-volume tertiary care health facility in Peshawar, Pakistan. Unlike other regional practices focusing solely on pain scores, our study examines the interplay of four key dimensions: medication adherence, pain trajectories, functional recovery, and system efficiency.

This study, therefore, seeks to evaluate the clinical and systemic outcomes of a standardized acetaminophen-based protocol at Lady Reading Hospital, addressing dimensions that extend beyond pain scores to encompass medication adherence, functional recovery, and discharge efficiency [7]. By anchoring analysis in prospectively collected clinical data, this study provides actionable insights for protocol refinement. It also underscores the necessity of adapting global guidelines to local resource realities, such as leveraging ibuprofen’s low cost and temperature stability in settings with unreliable refrigeration. Future interventions must address not just pharmacological gaps but also structural barriers (e.g., discharge coordination) and cultural perceptions of pain management.

## Materials and methods

Study design and setting

This prospective observational study was conducted at Lady Reading Hospital, Peshawar, a high-volume tertiary care hospital serving the people of Khyber Pakhtunkhwa, Pakistan. The investigation spanned 10 months (October 2024 to July 2025) to capture seasonal variations in surgical volumes. Adhering to the Strengthening the Reporting of Observational Studies in Epidemiology (STROBE) guidelines for observational studies [8], the design enabled real-time tracking of perioperative protocols without intervention, ensuring ecological validity. As per the updated guidelines (2019) developed by the American Academy of Otolaryngology - Head and Neck Surgery Foundation, evidence-based recommendations for the pre-, intra-, and postoperative care and management of children one to 18 years of age under consideration for tonsillectomy are provided. Clinicians should recommend ibuprofen, acetaminophen, or both for pain control after tonsillectomy [9].

Sample size

The target enrollment was 99 participants. An initial sample size of 384 was estimated using Cochran’s formula for an infinite population [10]. This was subsequently adjusted using the finite population correction method based on the expected number of eligible patients during the study period, yielding a minimum required sample of 92.

Given the prospective design and fixed study duration, a final sample of 99 consecutively eligible patients was included. The sample size was therefore determined pragmatically, reflecting the feasible number of cases within the study period rather than a formal power-based calculation. Similar sample sizes have been reported in single-center pediatric surgical studies in comparable settings [11].

Subjects

Participants were recruited from the hospital’s pediatric otolaryngology service using consecutive sampling [12]. Eligible participants included children aged seven to 14 years who were scheduled for elective tonsillectomy with or without adenoidectomy for indications such as recurrent tonsillitis or sleep-disordered breathing. Written informed consent was obtained from parents or guardians prior to enrollment.

Patients were excluded if they had a history of chronic pain syndromes (such as juvenile idiopathic arthritis), known hypersensitivity to acetaminophen or NSAIDs, or were undergoing concurrent complex surgical procedures such as cleft palate repair.

Data collection

Data were systematically collected using a structured proforma completed intraoperatively and throughout the 24-hour postoperative period. Trained nursing staff recorded: (1) demographics (age documented in full years); (2) perioperative medications, including intraoperative intravenous acetaminophen (15 mg/kg administered 30 minutes before incision) and postoperative oral acetaminophen (15 mg/kg every six hours) with optional pro re nata (PRN) ibuprofen (10 mg/kg oral; as needed); (3) While a baseline anesthesia induction was done through propofol (acc to body weight) muscle relaxation with atracurium (acc to body weight) maintenance with isoflurane, dexamethasone iv 0.1 mg/kg and tramadol 2 mg/kg. Pain assessment using a numeric scale (0-10) for children ≥8 years and the Faces Pain Rating Scale for children <8 years; and (4) Key outcome metrics: discharge status within 24 hours (coded as Yes/No), tolerance of cold liquids without vomiting (oral intake), and presence of pain at discharge (binary Yes/No assessment by nursing staff). Note on pain scale comparability: The NRS (used for children ≥8 years) and the Wong-Baker Faces Pain Rating Scale (used for children <8 years) differ in psychometric construct and response anchoring; direct numerical comparisons across age sub-groups should therefore be interpreted with caution [13]. Pain scores are reported separately by instrument in the results. Future studies should adopt a single age-validated instrument spanning the full study age range (e.g., the Faces Pain Scale-Revised, validated ages four to 16 years). All tonsillectomies were performed using a standardized cold dissection technique. Hemostasis was achieved primarily with suture ligation. Electrocautery was not used routinely and was reserved only for re-exploration cases requiring control of secondary hemorrhage.

Data analysis

Data were analyzed using SPSS version 25 (IBM Corp, Armonk, NY). Descriptive statistics generated frequencies and percentages for all categorical variables (age, medications, outcomes) [14]. Frequency distribution tables summarized cohort characteristics, while bar charts visualized pain score patterns and discharge trends across age strata. Data cleaning addressed minor entry errors (e.g., consolidating "N0" and "NO" in ibuprofen fields). The study received ethical approval with anonymized data stored as encrypted files on password-protected servers.

## Results

The cohort comprised 99 patients aged seven to 14 years, with no representation from ages five to six, 10, or 13 years. Age distribution analysis revealed two dominant groups: 12- and 14-year-olds each constituted 25.3% (n=25) of the cohort, reflecting peak tonsillectomy incidence in early adolescence. Nine-year-olds represented 20.2% (n=20), while eight-year-olds accounted for 11.1% (n=11). The smallest subgroup was seven-year-olds at 8.1% (n=8). This distribution underscores a clinical preference in the setting to avoid elective tonsillectomy in children under seven years due to anesthesia risks, as shown in Table 1.

**Table 1:** Demographic Profile and Age Distribution of Study Cohort (N=99)

Age (Years)	N	% of Cohort	Male, n (%)	Female, n (%)	Cumulative %
7	8	8.10%	5 (62.5%)	3 (37.5%)	8.10%
8	11	11.10%	7 (63.6%)	4 (36.4%)	19.20%
9	20	20.20%	12 (60.0%)	8 (40.0%)	39.40%
10	0	0.00%	-	-	39.40%
11	10	10.10%	6 (60.0%)	4 (40.0%)	49.50%
12	25	25.30%	15 (60.0%)	10 (40.0%)	74.70%
13	0	0.00%	-	-	74.70%
14	25	25.30%	14 (56.0%)	11 (44.0%)	100.00%
Total	99	100%	59 (59.6%)	40 (40.4%)	—

Medication adherence demonstrated high consistency in acetaminophen administration but revealed critical gaps in multimodal analgesia implementation. Intraoperative intravenous acetaminophen (15 mg/kg) was administered to 100% of patients, with 100% also receiving postoperative acetaminophen (15 mg/kg), confirming strict protocol compliance for this analgesic. Conversely, only six patients (6.1%) received ibuprofen, and the pain of these patients did not subside. Notably, data-cleaning procedures corrected documentation errors (e.g., "N0" entries revised to "NO"), ensuring reporting accuracy, as shown in Table 2.

**Table 2:** : Pain Management Medication

Medication	Frequency	Valid Percent %
Intra-op acetaminophen	99	100
Post-op acetaminophen	99	100
Ibuprofen administered	6	6.1

Key recovery metrics demonstrated effective pain management and functional recovery but revealed significant systemic inefficiencies in discharge processes. No patients (0%, n=0) reported pain at discharge, affirming acute pain management success. Functional recovery was robust, with 100% of patients tolerating cold liquids, indicating a rapid return to baseline oral function. However, only 73 patients (73.7%) were discharged within the target 24-hour window, while 26 patients (26.3%) remained hospitalized due to other surgical complications, such as bleeding or suspected bleeding, among other clinical or systemic issues that delayed discharge, despite meeting all clinical discharge criteria (absence of pain and tolerance of oral intake), as shown in Table 3.

**Table 3:** Postoperative Outcomes

Outcomes	Frequency	Valid Percent %
Discharge to home	73	73.7
Pain at discharge	0	0
Oral intake (Liquids)	99	100

Mild pain (score 3/10) was dominant, reported by 73.7% (n=73) of the patients, aligning with acetaminophen's established efficacy for low-intensity pain management. A moderate pain cohort (score 5/10) comprised 20.2% (n=20) of patients. This group was predominantly younger children aged seven to nine years (15/20, 75%) and consisted overwhelmingly of non-ibuprofen recipients (19/20, 95%), suggesting potential undertreatment in this subgroup. Additionally, 6.1% (n=6) of patients reported a transitional pain score of 4/10.

Analysis revealed significant age-linked disparities in discharge efficiency. High discharge rates were observed among eight-year-olds (100%, n=11/11), 14-year-olds (92%, n=23/25), and nine-year-olds (85%, n=17/20). In contrast, problematic retention emerged in specific cohorts: only 60% of 11-year-olds (n=6/10) were discharged within target timelines, with 40% (n=4) unnecessarily retained despite meeting criteria. Similarly, 24% of 12-year-olds (n=6/25) experienced delays despite 76% (n=19/25) being discharged promptly. This demonstrates systemic inefficiencies disproportionately affecting early adolescents.

## Discussion

Reinforcing acetaminophen's role in pain management

The universal adherence to acetaminophen demonstrates its viability as a foundational analgesic in resource-constrained settings [9]. This consistency directly aligned with global guidelines and yielded two critical successes: 73.7% of patients reported only mild pain (score=3 on the 0-10 pain scale), and all achieved oral intake tolerance, a key predictor of recovery speed and reduced dehydration risk [15]. These outcomes mirror multicenter studies where scheduled acetaminophen reduced rescue opioid needs by 40% in pediatric tonsillectomies [16]. However, this study reveals acetaminophen's limitations as monotherapy. Pharmacodynamic studies show children aged seven to nine years metabolize acetaminophen 25% faster than adolescents due to immature hepatic enzymes, explaining why this subgroup constituted 75% of moderate pain cases (score=5 on the 0-10 pain scale). This underscores the need for age-stratified dosing rather than the fixed 10 mg/kg protocol applied uniformly across seven- to 14-year-olds.

The multimodal gap: ibuprofen underutilization and its consequences

The negligible ibuprofen administration (6.1%) represents a significant departure from evidence-based practice [9]. It is important to clarify that the postoperative analgesic protocol in this study constitutes acetaminophen monotherapy with PRN ibuprofen as rescue analgesia, not true multimodal analgesia as defined by evidence-based guidelines (which require the systematic, concurrent use of two or more agents via different pharmacological mechanisms). The intraoperative use of dexamethasone and tramadol does represent intraoperative multimodal elements; however, these are distinct from the postoperative protocol under evaluation. Future protocols should incorporate scheduled acetaminophen-ibuprofen combination as the standard postoperative regimen to fulfil the criteria for true multimodal analgesia. Global consensus guidelines explicitly recommend NSAID-acetaminophen combinations as first-line therapy [11], with meta-analyses confirming a 35% reduction in moderate-to-severe pain versus acetaminophen alone [16]. This underuse likely stems from persistent, unfounded fears of post-tonsillectomy bleeding incidence of PTH increased significantly between 2011 and 2015, and ibuprofen appears to be one contributing factor [17]. In our cohort, 95% of patients reporting moderate pain (score=5 on the 0-10 pain scale) received no ibuprofen, suggesting this gap directly compromised pain control. Crucially, ibuprofen’s role in a significant reduction in the number of postoperative doses and the amount of fentanyl administered after surgery in the IV-ibuprofen group compared with the placebo group (P=0.021), hinting a better synergy with acetaminophen in reducing the use of opioids [18].

Deciphering systemic barriers

Despite a higher discharge ratio in our study, the retention of 26.3% patients was due to other surgical complications such as bleeding or suspected bleeding, among other clinical or systemic issues that delayed discharge, despite clinical readiness (no pain, tolerating liquids). By clustering together non-discharge in 11-year-olds (40%) and 12-year-olds (24%) hints age-specific social dynamics [19]. Key barriers to timely discharge identified in the study include parental anxiety, particularly for children aged 10-12 years who are perceived as "too old for pediatric care but too young for adult resilience," heightening fears of complications. Logistical delays, such as missing discharge paperwork or transportation issues, disproportionately affect early adolescents who often present unaccompanied [20]. Additionally, insurance authorization requirements for extended stays (10+ beds) contribute to bureaucratic delays [21]. These non-clinical factors extend hospital stays unnecessarily, increasing costs without improving outcomes.

Limitations: contextualizing generalizability and protocol rigidity

The absence of five- to six-year-olds - a high-pain-risk cohort - limits protocol applicability to younger children. This gap reflects real-world constraints: in many low- and middle-income countries (LMIC), there is parental anxiety for under-seven children who have undergone surgeries [20]. Similarly, the homogeneous acetaminophen dosing prevented dose-response analysis. Future studies should incorporate weight-tiered regimens (e.g., 15 mg/kg for <25 kg) [7] and track plasma concentrations to optimize therapeutic efficacy. The use of two distinct pain assessment tools (NRS for children ≥8 years and Wong-Baker Faces for children <8 years) limits direct comparability of pain scores across age sub-groups, as these instruments differ in psychometric properties and construct validity. Future studies should employ a single age-validated instrument spanning the full pediatric age range, such as the Faces Pain Scale - Revised (FPS-R), to enable uniform outcome analysis and improve inter-centre reproducibility. Additionally, the discharge pain field was a binary nursing judgment (YES/NO) rather than a validated serial pain score at predefined time points; the absence of T0-T24 serial assessments precluded repeated measures analysis in the present study. This is acknowledged as a key analytical limitation; the revised data collection proforma now incorporates standardized T0, T2, T4, T6, T8, and T24 pain assessments to enable linear mixed-effects model (LMEM) analysis in future iterations.

Recommendations: from evidence to practice

To address identified gaps, the study proposes three key recommendations: First, revision of medication protocols to include mandatory ibuprofen (10 mg/kg) administered eight-hourly for 48 hours in children over seven years without contraindications [7]. Second, implementation of discharge efficiency measures, such as structured preoperative parent education to reduce anxiety-driven retention [20], and dedicated discharge coordinators to expedite paperwork and transportation logistics for 10- to 14-year-olds. Third, adoption of an implementation science approach to pilot revised protocols in high-retention subgroups (11- to 12-year-olds), using pain scores and discharge lag times as key performance indicators [21].

## Conclusions

This study demonstrates that standardized perioperative acetaminophen provides effective baseline pain control and supports safe recovery in pediatric tonsillectomy patients in a resource-limited setting. However, the limited use of ibuprofen highlights an important gap in the implementation of multimodal analgesia, particularly in younger children who may experience higher pain levels.

These findings emphasize the need for integrating evidence-based multimodal pain management strategies, including routine use of NSAIDs, along with age-appropriate dosing approaches. Additionally, addressing non-clinical factors such as discharge processes and caregiver concerns is essential to improve overall patient outcomes. Optimizing both pharmacological and system-level practices will be critical in enhancing postoperative care and ensuring efficient recovery in similar healthcare settings.
